# Protective Effects of Licochalcone A Improve Airway Hyper-Responsiveness and Oxidative Stress in a Mouse Model of Asthma

**DOI:** 10.3390/cells8060617

**Published:** 2019-06-20

**Authors:** Wen-Chung Huang, Chien-Yu Liu, Szu-Chuan Shen, Li-Chen Chen, Kuo-Wei Yeh, Shih-Hai Liu, Chian-Jiun Liou

**Affiliations:** 1 Graduate Institute of Health Industry Technology, Research Center for Food and Cosmetic Safety, Research Center for Chinese Herbal Medicine, College of Human Ecology, Chang Gung University of Science and Technology, No.261, Wenhua 1st Rd., Guishan Dist., Taoyuan City 33303, Taiwan; wchuang@mail.cgust.edu.tw (W.-C.H.); cyliu01@mail.cgust.edu.tw (C.-Y.L.); 2 Division of Allergy, Asthma, and Rheumatology, Department of Pediatrics, Chang Gung Memorial Hospital, Linkou, Guishan Dist., Taoyuan City 33303, Taiwan; lcchen@cgmh.org.tw (L.-C.C.); kwyeh@cgmh.org.tw (K.-W.Y.); 3 Graduate Program of Nutrition Science, National Taiwan Normal University, 88 Ting-Chow Rd, Sec 4, Taipei 11677, Taiwan; scs@ntnu.edu.tw; 4 School of Medicine, Taipei Medical University, No. 250, Wu-Hsing Street, Taipei 110, Taiwan; liushihhao1295@gmail.com; 5 Department of Nursing, Division of Basic Medical Sciences, Research Center for Chinese Herbal Medicine, and Graduate Institute of Health Industry Technology, Chang Gung University of Science and Technology, No.261, Wenhua 1st Rd., Guishan Dist., Taoyuan City 33303, Taiwan

**Keywords:** airway hyper-responsiveness, asthma, eosinophil, oxidative stress, licochalcone A

## Abstract

Licochalcone A was isolated from *Glycyrrhiza uralensis* and previously reported to have antitumor and anti-inflammatory effects. Licochalcone A has also been found to inhibit the levels of Th2-associated cytokines in the bronchoalveolar lavage fluid (BALF) of asthmatic mice. However, the molecular mechanism underlying airway inflammation and how licochalcone A regulates oxidative stress in asthmatic mice are elusive. In this study, we investigated whether licochalcone A could attenuate inflammatory and oxidative responses in tracheal epithelial cells, and whether it could ameliorate oxidative stress and airway inflammation in asthmatic mice. Inflammatory human tracheal epithelial (BEAS-2B) cells were treated with licochalcone A to evaluate oxidative responses and inflammatory cytokine levels. In addition, BALB/c mice were sensitized with ovalbumin (OVA) and injected intraperitoneally with licochalcone A (5 or 10 mg/kg). Licochalcone A significantly inhibited reactive oxygen species, eotaxin, and proinflammatory cytokines in BEAS-2B cells. Licochalcone A also decreased intercellular adhesion molecule 1 levels in inflammatory BEAS-2B cells, blocking monocyte cell adherence. We also found that licochalcone A significantly decreased oxidative responses, reduced malondialdehyde levels, and increased glutathione levels in the lungs of OVA-sensitized mice. Furthermore, licochalcone A decreased airway hyper-responsiveness, eosinophil infiltration, and Th2 cytokine production in the BALF. These findings suggest that licochalcone A alleviates oxidative stress, inflammation, and pathological changes by inhibiting Th2-associated cytokines in asthmatic mice and human tracheal epithelial cells. Thus, licochalcone A demonstrated therapeutic potential for improving asthma.

## 1. Introduction

Asthma is an important health problem, with continually increasing prevalence and mortality in developing and developed countries [1,2]. Allergic asthma is a chronic airway inflammatory disease characterized by contained paroxysmal wheezing, dry cough, shortness of breath, and chest tightness [3]. Pathological studies in asthmatic patients have demonstrated airway smooth muscle proliferation that causes airway narrowing and goblet cell hyperplasia that increases mucus hypersecretion, blocking the airways [4]. A sudden asthma attack would cause airway hyper-responsiveness (AHR) and airflow obstruction, even leading to difficulty breathing and death [2].

Several studies have demonstrated that allergic asthma is an immune imbalance in which overactivation of Th2 cells increases the secretion of IL-4, IL-5, and IL-13 cytokines [5]. These Th2-associated cytokines would induce eosinophil infiltration, inducing inflammation and the allergy response, stimulate goblet cell hyperplasia for excessive mucus secretion, and exacerbate IgE production to induce mast cell activation and cause a severe allergic reaction [6]. Inflammatory immune cells, including neutrophils, monocytes, and eosinophils, could increase the levels of reactive oxygen species (ROS) to exacerbate inflammation and injury in lung tissue [7]. Recent research shows that activated eosinophils release excessive eosinophil peroxidase and eosinophil cationic protein, causing an allergic, inflammatory reaction and ROS in the lung tissue [8]. Moreover, inflammatory respiratory epithelial cells release high levels of chemokines for attracting eosinophilic infiltration to aggravate airway inflammation [1,9]. These epithelial cells also release excessive ROS, inducing airway remodeling, abnormal smooth muscle thickness, and AHR [10]. ROS and inflammation were confirmed as the key deleterious signals for the development of asthma symptoms [4,11]. Thus, it was an important ameliorated strategy that regulated the activity of Th2 cells and decreased inflammation and ROS-associated signals in asthma progression.

Licochalcone A is a chalcone isolated from *Glycyrrhiza uralensis* Fisch [12]. Licochalcone A has multiple biological functions in cellular and animal models, and it has been demonstrated to reduce the inflammatory response in lipopolysaccharide (LPS)-stimulated macrophages and induce apoptosis and autophagy in tumor cells [13,14,15,16]. Licochalcone A could also decrease ROS, promoting neuroprotective effects [17]. Recently, licochalcone A was found to attenuate airway inflammation in ovalbumin (OVA)-sensitized mice [18], but whether it improves AHR and oxidative stress is still unclear. In the current study, we evaluated whether licochalcone A ameliorates the molecular mechanisms of airway inflammatory and oxidative stress in asthmatic mice. We also evaluated whether licochalcone A modulates oxidative responses and inflammatory cytokine levels in inflammatory human tracheal epithelial (BEAS-2B) cells.

## 2. Materials and Methods

### 2.1. Animals

Six-week-old female BALB/c mice were purchased from the National Laboratory Animal Center in Taiwan and raised in air-conditioned animal housing with food and water ad libitum. Animal experiments were approved by the Laboratory Animal Care Committee of Chang Gung University of Science and Technology (IACUC approval number: 2018-004). Licochalcone A (≥98% purity by HPLC; Sigma-Aldrich, St. Louis, MO, USA) was dissolved in dimethyl sulfoxide (DMSO). Mice were divided into 4 experimental groups of 12 animals each: normal control mice (N group), mice were sensitized with normal saline and treated with DMSO by intraperitoneal injection; OVA-sensitized control mice (OVA group), mice were sensitized with OVA and treated with DMSO by intraperitoneal injection; and LA5 and LA10 groups, OVA-sensitized mice were treated with 5 or 10 mg/kg licochalcone A, respectively.

### 2.2. Sensitization and Administration of Licochalcone A

**Figure 1:**
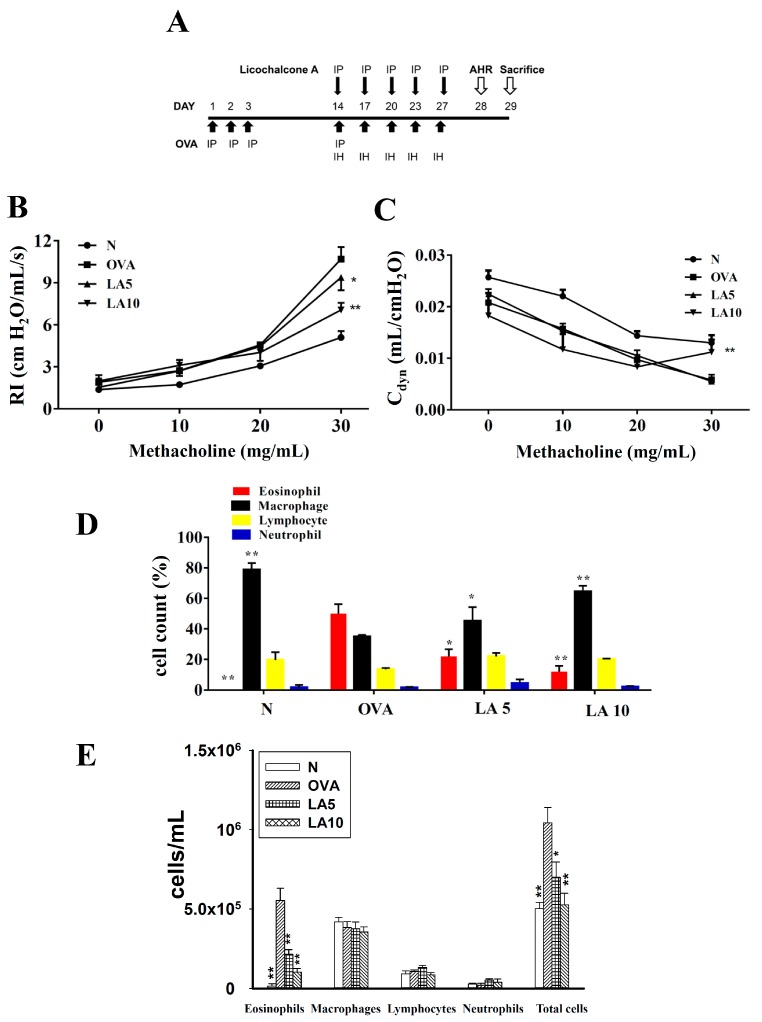
The effect of licochalcone A (LA) on airway hyper-responsiveness (AHR) and cell counts in bronchoalveolar lavage fluid (BALF) of asthmatic mice. (**A**) On days 1–3 and 14, mice were sensitized with ovalbumin (OVA) by intraperitoneal injection (IP) and challenged with 2% OVA inhalation (IH) on days 14, 17, 20, 23, and 27. One hour before the OVA challenge or methacholine inhalation, mice were treated with LA or DMSO (*n* = 12 mice/group). (**B**) AHR was measured as a percentage of lung resistance (RI) from baseline normal (N) and (**C**) dynamic lung compliance (C_dyn_). (**D**) Inflammatory cells were measured and the percentage of inflammatory cells in the BALF presented. (**E**) Inflammatory cells and total cells were measured in BALF. Three independent experiments were analyzed, and data were presented as mean ± SEM. * *P* < 0.05 compared to the OVA control group. ** *P* < 0.01 compared to the OVA control group.

Mice were sensitized as shown in Figure 1A and as described previously [19]. Briefly, mice were treated with 200 μL of the sensitization solution containing 50 μg OVA (Sigma) and 0.8 mg aluminum hydroxide (Thermo, Rockford, IL, USA) in normal saline by intraperitoneal injections on days 1–3 and 14. Next, mice were challenged with inhaled 2% OVA for 30 min on days 14, 17, 20, 23, and 27 using an ultrasonic nebulizer (DeVilbiss Pulmo-Aide 5650D, Drive DeVilbiss International, Port Washington, NY, USA) with a nebulization rate of 0.15–0.35 mL/min and aerosolized particle size of 0.5 to 5 μm. The mice were injected intraperitoneally with DMSO or licochalcone A 1 h before OVA challenge or methacholine (Sigma) inhalation (day 28). AHR was assessed on day 28, and mice were sacrificed to evaluate asthma pathology, oxidative pressure, immune regulation, and inflammatory response on day 29.

### 2.3. Airway Hyper-Responsiveness (AHR)

Airway function was demonstrated using aerosolized methacholine as described previously [20]. The mice were also anesthetized and intubated to measure respiratory resistance and dynamic lung compliance using a low-frequency, forced oscillation technique (Buxco Electronics, Troy, NY, USA) as described previously [21].

### 2.4. Bronchoalveolar Lavage Fluid (BALF) and Cell Counting

Mice were sacrificed and bronchoalveolar lavage fluid (BALF) collected as described previously [22,23]. The trachea was intubated using an indwelling needle to wash the lungs and airways with 1 mL of normal saline. The BALF was centrifuged at 1500 rpm for 5 min and supernatant collected to detect the levels of cytokines and chemokines. The cells were stained with Giemsa stain (Sigma) to differentiate cell morphology and to determine the percentages of immune cells.

### 2.5. Histological Analysis of Lung Tissue

Lung tissues were removed and fixed with formalin before embedding in paraffin. The blocks were cut into six-micrometer-thick sections for hematoxylin and eosin (HE) staining to evaluate eosinophil infiltration using a five-point scoring system [24]. The degree of cell infiltration was scored as follows: 0, no cells; 1, a few cells; 2, a ring of inflammatory cells, one cell layer; 3, a ring of inflammatory cells, two to four cell layers; and 4, a ring of inflammatory cells, more than four cell layers. In addition, sections were stained with Masson’s trichrome stain to observe collagen expression and periodic acid–Schiff (PAS) stain (Sigma) to detect goblet cell hyperplasia [20,25]. PAS-positive cells were expressed per 100 μm of basement membrane.

### 2.6. Immunofluorescence Staining

Lung tissues were embedded in paraffin, and the section was incubated with primary antibody and then fluorescent secondary antibodies. The slide was washed with 4’,6-diamidino-2-phenylindole (DAPI) for nuclear staining and observed under a fluorescence microscope (Olympus). Primary antibodies included γH2AX and clara cell 10 (CC10) (Cell Signaling Technology, Danvers, MA, USA).

### 2.7. Glutathione (GSH) Assay

A glutathione assay kit (Sigma) was used to detect the levels of glutathione in lung tissues according to the manufacturer’s instructions [22]. Lung tissues were placed in 5% 5-sulfosalicylic acid solution and homogenized using a homogenizer (FastPrep-24, MP Biomedicals, Santa Ana, CA, USA). The samples were centrifuged at 10,000× *g* for 10 min, and glutathione levels were determined at an optical density (OD) of 412 nm using a microplate reader (Multiskan FC, Thermo, Waltham, MA, USA).

### 2.8. Malondialdehyde (MDA) Activity

A lipid peroxidation assay kit (Sigma) was used to detect malondialdehyde (MDA) activity in the lungs according to the manufacturer’s instructions [26]. Lung tissues were homogenized and treated with perchloric acid for protein precipitation. The samples were centrifuged at 10,000× *g* for 10 min, and MDA activity was detected in the supernatant using a multimode microplate reader (BioTek SynergyHT, Bedfordshire, UK).

### 2.9. RNA Isolation and Quantitative Real-Time PCR Analysis

RNA was extracted from lung tissue using TRI reagent (Sigma), and complementary DNA (cDNA) was synthesized using a cDNA synthesis kit (Bio-Rad, San Francisco, CA, USA). The expressions of specific genes were determined using SYBR Green Master Mix (Bio-Rad) for quantitative real-time PCR. For gene amplification, samples were preincubated at 95 °C for 10 min, followed by 40 cycles of 95 °C for 15 s and 60 °C for 1 min using a spectrofluorometric thermal cycler (iCycler; Bio-Rad). Expression was quantified using the ^∆∆^Ct method. The primers used for quantitative real-time PCR are given in Table 1.

### 2.10. Western Immunoblot Analysis

Lung tissues were homogenized and proteins separated on 10% SDS polyacrylamide gels. The proteins were transferred into polyvinylidene difluoride membranes and incubated with primary antibodies at 4 °C. The next day, the membranes were incubated with secondary antibodies and treated with Luminol/Enhancer Solution (Millipore) to obtain specific protein signals using the BioSpectrum 600 system (UVP, Upland, CA, USA). Primary antibodies included COX-2, HO-1, Nrf2, Lamin B1 (Santa Cruz Biotechnology Inc., Santa Cruz, CA, USA), and β-actin (Sigma).

### 2.11. Serum Collection

Blood was centrifuged at 6000 rpm for 5 min, serum collected, and stored at −80 °C. OVA-specific antibodies were measured by enzyme-linked immunosorbent assay (ELISA) as described previously [27].

### 2.12. ELISA

BALF and cell culture supernatant were used to detect the levels of intercellular adhesion molecule 1 (ICAM-1), IL-6, TNF-α, CCL5, CCL11, CCL24, MCP-1, IL-4, IL-5, IL-8, and IL-13 using specific ELISA kits according to the manufacturer’s instructions (R&D Systems, Minneapolis, MN, USA) [27,28]. The serum levels of OVA-specific IgG1 and IgE were investigated using a specific ELISA kit (BD Biosciences, San Jose, CA, USA). Serum was diluted five-fold to demonstrate the absorbance of OVA-IgE at an OD of 450 nm. OVA-IgG1 standard curves were obtained using serum from OVA-sensitized mice.

### 2.13. Biochemical Analysis of Serum

The serum levels of glutamate oxaloacetate transaminase (GOT) and glutamic pyruvic transaminase (GPT) were detected using a biochemical analyzer (DRI-CHEM NX500, Fuji, Tokyo, Japan).

### 2.14. BEAS-2B Cell Culture and Licochalcone A Treatment

Licochalcone A was dissolved in DMSO, and a 30 mM stock solution was made. In the experimental culture medium, DMSO was <0.1%. BEAS-2B cells (American Type Culture Collection, Manassas, VA, USA) were seeded onto 24-well plates in DMEM/F12 medium. The cells were treated with licochalcone A (0–20 μM) for 1 h, then stimulated with 10 ng/mL TNF-α and 10 ng/mL IL-4 for 24 h. The supernatants were used to detect the chemokine or cytokine levels using specific ELISA kits.

### 2.15. Determination of ROS Production

BEAS-2B cells were treated with licochalcone A for 1 h, and then they were stimulated with TNF-α and IL-4 for 24 h. Next, the cells were incubated with 20 μM 2′,7′-dichlorofluorescin diacetate (DCFH-DA) for 30 min. Cells were lysed and ROS levels detected using a multimode microplate reader (BioTek synergy HT) with excitation at 485 nm and emission at 528 nm as described previously [21,29]. Moreover, intracellular ROS were observed by fluorescence microscopy (Olympus).

### 2.16. Cell–Cell Adhesion Assay

BEAS-2B cells were treated with licochalcone A and stimulated with TNF-α/IL-4 for 24 h. THP-1 human monocytic cells were obtained from the Bioresource Collection and Research Center (BCRC, Taiwan) and cultured in RPMI 1640 medium supplemented with 10% FBS and 100 U/mL penicillin and streptomycin. THP-1 cells were stained with calcein-AM solution (Sigma) for 30 min. BEAS-2B cells were co-cultured with THP-1 cells, and adherent THP-1 cells were observed by fluorescence microscopy (Olympus, Tokyo, Japan) as described previously [30].

### 2.17. Statistical Analysis

Statistical analyses were performed using one-way analysis of variance (ANOVA) and a Dunnett post hoc test. Data were expressed as the mean ± SEM of at least three independent experiments. *P* < 0.05 was considered significant.

## 3. Results

### 3.1. Licochalcone A Attenuated AHR in Asthmatic Mice

First, we evaluated whether licochalcone A improved abnormal airflow in the airways of asthmatic mice. Airway resistance as detected by the forced intubation technique demonstrated that 10 mg/kg licochalcone A significantly attenuated airway resistance and increased dynamic lung compliance compared to OVA (Figure 1B,C).

### 3.2. Licochalcone A Reduced Eosinophils in BALF

We counted the proportion of inflammatory cells in BALF to assess whether licochalcone A decreased the inflammatory response in asthmatic mice. Asthmatic mice treated with licochalcone A had a significantly reduced proportion of eosinophils compared to the OVA group (LA5: 21.9% ± 4.8%, *P* < 0.05; LA10: 11.9% ± 3.8%, *P* < 0.01 vs. OVA: 50.1% ± 6.1%) (Figure 1D). Compared to the OVA group, asthmatic mice treated with licochalcone A had significantly reduced numbers of eosinophils and total cells (Figure 1E).

### 3.3. Licochalcone A Suppressed Eosinophil Infiltration and Goblet Cell Hyperplasia in the Lungs

**Figure 2:**
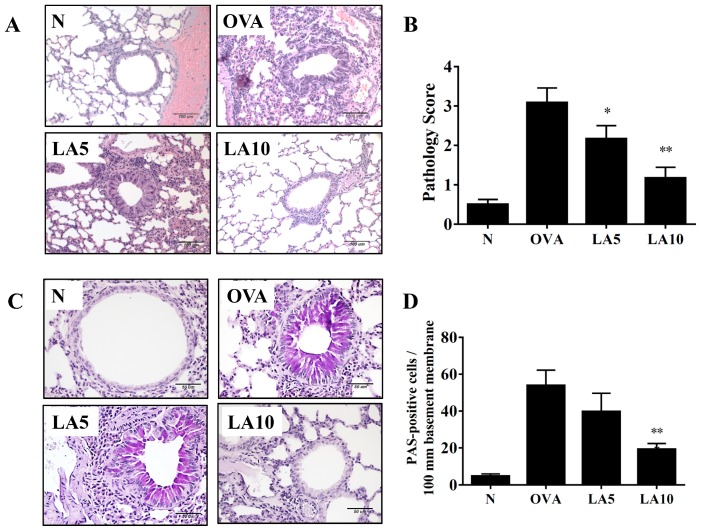
Effects of licochalcone A (LA) on asthmatic lung tissue. Histological sections of lung tissues from normal (N) and OVA-stimulated (OVA) mice with or without LA (LA5 and LA10) treatment. (**A**) LA reduced eosinophil infiltration (HE stain; 200× magnification) and (**B**) scoring of inflammation in pathological evaluation of inflammatory cell infiltration in lung sections. (**C**) Periodic acid–Schiff (PAS)-stained lung sections demonstrated goblet cell hyperplasia. Goblet cells are indicated with arrows (200× magnification). (**D**) Results were expressed as the number of PAS-positive cells per 100 μm of basement membrane. Three independent experiments were analyzed, and data were presented as mean ± SEM. * *P* < 0.05 compared to the OVA control group. ** *P* < 0.01 compared to the OVA control group.

In asthmatic lungs, a large infiltration of inflammatory cells could cause an allergic and inflammatory response [31]. OVA-sensitive mice had more eosinophil infiltration between the bronchus and blood vessels than normal control mice. OVA-sensitized mice treated with licochalcone A had inhibited eosinophil infiltration in the lungs, and the inflammatory pathology score showed that licochalcone A significantly decreased inflammatory pathology in the lungs of asthmatic mice (Figure 2A,B). Evaluating tracheal goblet cell hyperplasia using PAS staining, numerous inflammatory goblet cells were expressed in the bronchus in OVA-sensitized mice, and licochalcone A significantly decreased goblet cell hyperplasia compared to the OVA group (Figure 2C,D).

### 3.4. Licochalcone A Reduced Collagen and COX-2 Expression in the Lungs

**Figure 3:**
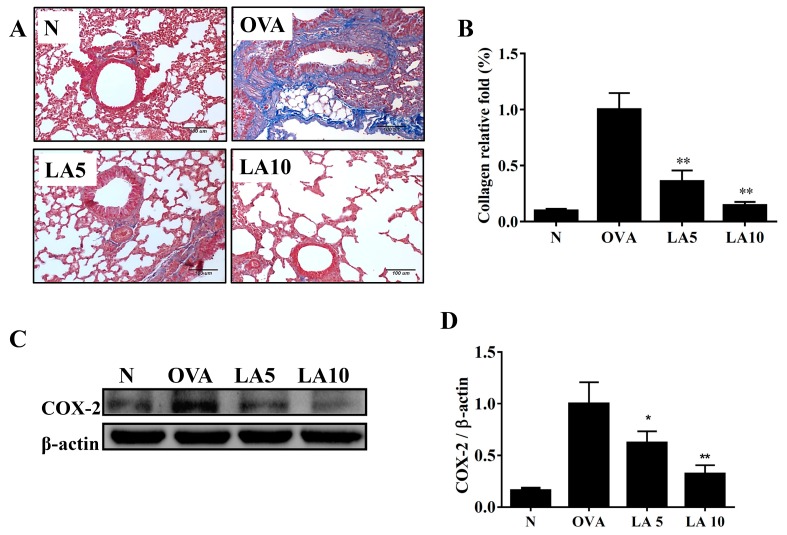
Effects of licochalcone A (LA) on collagen expression in lung tissue from OVA-sensitized mice. (**A**) Lung sections were stained with Masson’s trichrome stain to detect collage expression (200× magnification). (**B**) Quantitative analysis of collagen in lung sections. (**C**) Western blot showing that LA suppressed COX-2 expression in lung tissue. (**D**) The fold-change in COX-2 protein expression was measured relative to the expression of β-actin. Three independent experiments were analyzed, and data were presented as mean ± SEM. * *P* < 0.05 compared to the OVA control group. ** *P* < 0.01 compared to the OVA control group.

Collagen production in the lungs was detected by Masson’s trichrome stain, and licochalcone A significantly reduced collagen expression in the lung tissue (Figure 3A,B). Licochalcone A also decreased COX-2 expression compared to the OVA group (Figure 3C,D).

### 3.5. Licochalcone A Modulated Cytokine and Chemokine Levels in Lung Tissue and BALF

**Figure 4:**
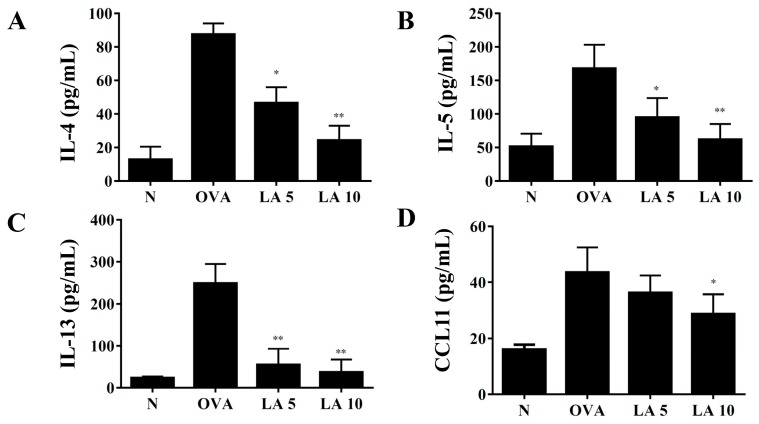
Effects of licochalcone A (LA) on the levels of cytokines and chemokines in BALF. The concentrations of (**A**) IL-4, (**B**) IL-5, (**C**) IL-13, and (**D**) CCL11 in BALF from normal (N) and OVA-stimulated (OVA) mice with or without licochalcone A (LA5-10) treatment were measured by ELISA. Three independent experiments were analyzed, and data were presented as mean ± SEM. * *P* < 0.05 compared to the OVA control group. ** *P* < 0.01 compared to the OVA control group.

**Figure 5:**
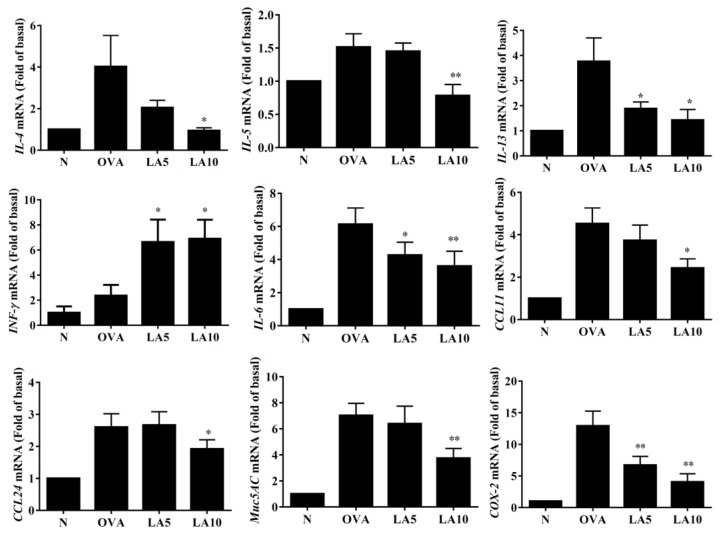
Effects of licochalcone A (LA) on the mRNA levels of cytokines, chemokines, and inflammatory mediators in the lungs. Gene expression levels were determined by real-time RT-PCR using RNA extracted from the lung tissues of normal (N) and OVA-stimulated (OVA) mice, with or without licochalcone A (LA5-10) treatment. Fold-changes in expression were measured relative to the *β-actin* expression (internal control). Three independent experiments were analyzed, and data were presented as mean ± SEM. * *P* < 0.05 compared to the OVA control group. ** *P* < 0.01 compared to the OVA control group.

In BALF, licochalcone A significantly suppressed IL-4, IL-5, IL-13, and CCL11 levels compared to OVA-sensitized mice. (Figure 4). We also detected gene expression in lung tissue, finding that licochalcone A significantly suppressed the levels of *IL-4, IL-5,* and *IL-13* expression compared to OVA-sensitized asthmatic mice. Furthermore, licochalcone A significantly suppressed *IL-6*, *COX-2*, *CCL11*, *CCL-24*, and *MUC5AC* expression compared to OVA-sensitized asthmatic mice and significantly increased *IFN-γ* production compared to asthmatic mice (Figure 5).

### 3.6. Licochalcone A Modulated GSH and MDA Activity in the Lungs

**Figure 6:**
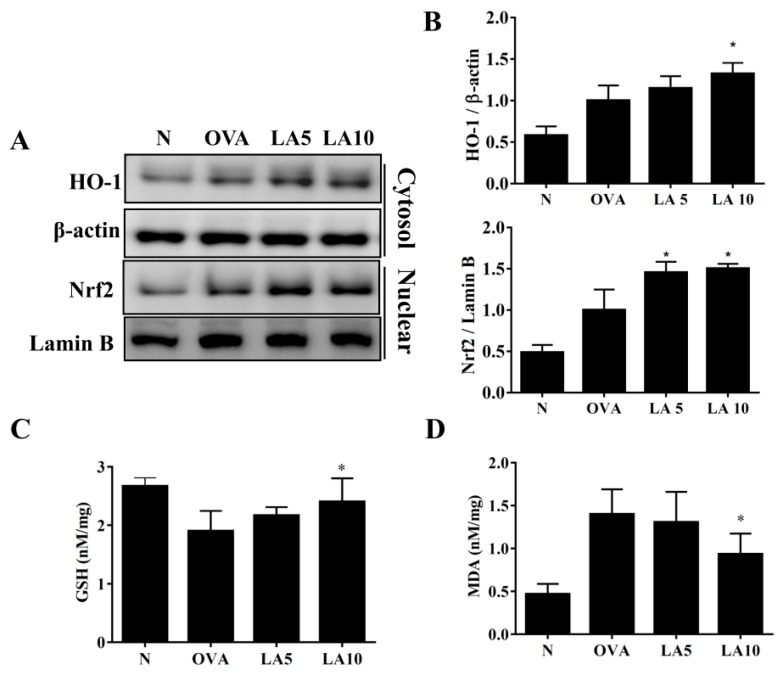
Effects of licochalcone A (LA) on oxidative stress factors. (**A**) Western blot showing LA modulation of HO-1 and Nrf2 expression in lung tissue from normal (N) and OVA-stimulated (OVA) mice with or without licochalcone A (LA5-10) treatment. (**B**) The fold-change in the expression of HO-1 and Nrf2 protein was measured relative to the expression of β-actin and lamin B, respectively. (**C**) Glutathione (GSH) activity and (**D**) malondialdehyde (MDA) activity in mouse lung tissue. Three independent experiments were analyzed, and data were presented as mean ± SEM. * *P* < 0.05 compared to the OVA control group. ** *P* < 0.01 compared to the OVA control group.

Continuous asthma attacks cause oxidative stress in the lungs [32]. Previous studies demonstrated that HO-1 has an antioxidative stress effect, decreasing lung damage [33]. Licochalcone A-treated asthmatic mice have increased HO-1 production in the lungs compared to OVA-sensitized asthmatic mice. In addition, licochalcone A promotes nuclear Nrf2 expression in the lungs of OVA-sensitized mice (Figure 6A,B). Furthermore, licochalcone A significantly decreased MDA activity and increased glutathione (GSH) levels in the lung tissue compared to OVA-sensitized asthmatic mice (Figure 6C,D).

### 3.7. Licochalcone A Did Not Improve DNA Damage in Ovalbumin (OVA)-Sensitized Asthmatic Mice

**Figure 7:**
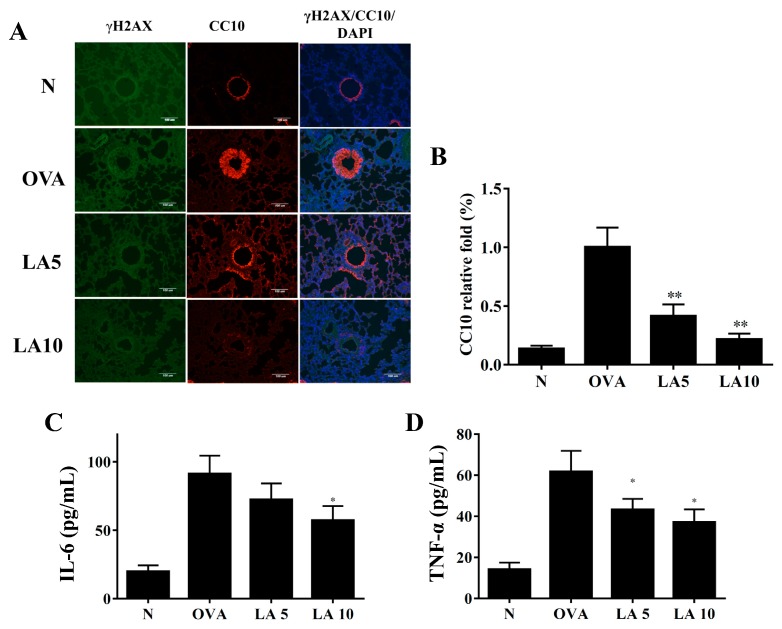
Licochalcone A (LA) affected DNA damage in asthmatic lung tissue. (**A**) Histological sections of lung tissues were stained for γH2AX (green) and CC10 (red) by immunofluorescence. Nuclei were stained with DAPI (blue) and observed by fluorescence microscopy. (**B**) Fold-changes in the expression levels of CC10 were measured and compared to the OVA group. Effects of LA on the levels of proinflammatory cytokines in BALF. (**C**) The concentrations of IL-6 and (**D**) TNF-α in BALF were measured by ELISA. Three independent experiments were analyzed, and data were presented as mean ± SEM. * *P* < 0.05 compared to the OVA control group. ** *P* < 0.01 compared to the OVA control group.

Acute allergic asthma stimulates inflammatory and oxidative stress, causing cell damage in lung tissue [34,35]. Previous studies demonstrated that house dust mites cause DNA double-strand breaks in the lungs of asthmatic mice [36]. Unfortunately, we did not detect γH2AX, the marker of DNA double-strand breaks, in the OVA-sensitized asthmatic mice (Figure 7A). Therefore, our result did not clearly confirm that licochalcone A ameliorated DNA double-strand breaks in this experimental asthma model. Interestingly, we found that CC-10, an anti-inflammatory protein, was significantly expressed in OVA-sensitized asthmatic mice, and licochalcone A suppressed CC-10 expression in the tracheas of asthmatic mice (Figure 7A,B). In BALF, licochalcone A significantly suppressed proinflammatory cytokines IL-6 and TNF-α compared to OVA-sensitized mice (Figure 7C,D).

### 3.8. Licochalcone A Modulated Serum OVA-Specific Antibody

**Figure 8:**
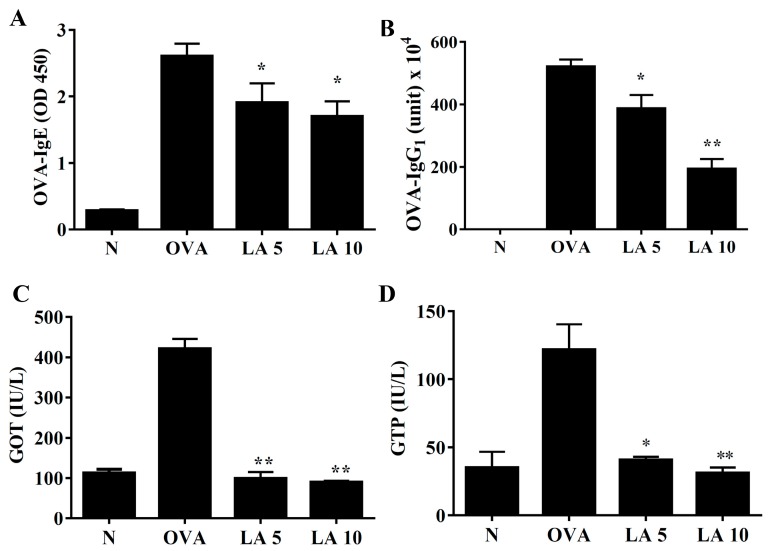
Effects of licochalcone A (LA) on OVA-specific antibodies in serum. (**A**) Serum levels of OVA-IgE and (**B**) OVA-IgG1 in normal (N) and OVA-stimulated (OVA) mice with or without licochalcone A (LA5-10) treatment. (**C**) Effects of LA on the serum biochemical value, including glutamate oxaloacetate transaminase (GOT) and (**D**) glutamic pyruvic transaminase (GPT). Three independent experiments were analyzed, and data were presented as mean ± SEM. * *P* < 0.05 compared to the OVA control group. ** *P* < 0.01 compared to the OVA control group.

In serum, licochalcone A significantly reduced the levels of OVA-IgG1 and OVA-IgE in OVA-sensitized asthmatic mice (Figure 8A,B). Licochalcone A also decreased glutamate oxaloacetate transaminase (GOT) and glutamic pyruvic transaminase GTP levels in asthmatic mice (Figure 8C,D).

### 3.9. Licochalcone A Suppressed Inflammatory Mediators and Cell Adhesion in BEAS-2B Cells

**Figure 9:**
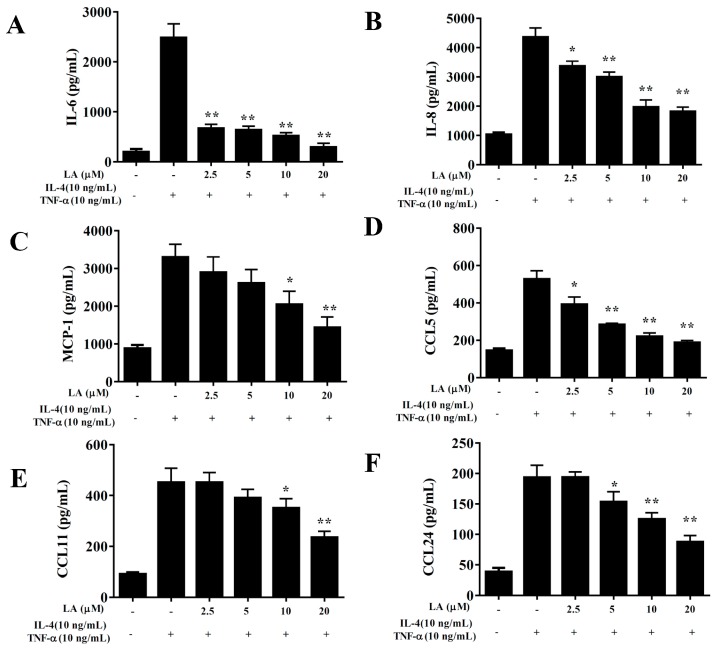
Effects of licochalcone A (LA) on cytokine and chemokine production in BEAS-2B cells. ELISA shows (**A**) IL-6, (**B**) IL-8, (**C**) MCP-1, (**D**) CCL5, (**E**) CCL11, and (**F**) CCL24 levels in BEAS-2B cells treated with TNF-α, IL-4, and/or LA. Data are presented as mean ± SEM. * *P* < 0.05, ** *P* < 0.01 compared to BEAS-2B cells stimulated with TNF-α and IL-4. Three independent experiments were analyzed and compared to TNF-α and IL-4.

**Figure 10:**
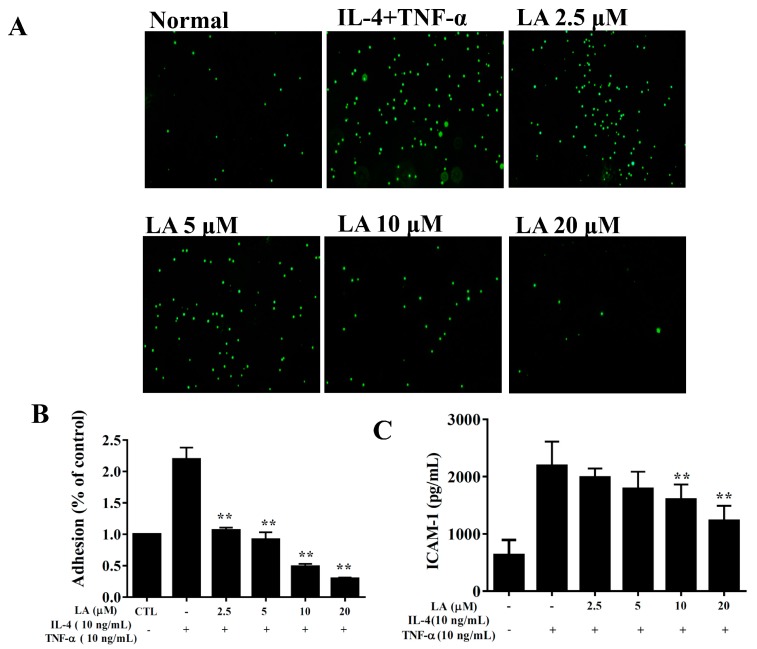
Licochalcone A (LA) inhibited THP-1 cell adherence to activated BEAS-2B cells. (**A**) Fluorescence microscopy images of THP-1 cells labeled with calcein-AM and mixed with normal (N) and TNF-α/IL-4-activated BEAS-2B cells in the absence or presence of LA. (**B**) Fluorescence intensity of monocytic cell adhesion to BEAS-2B cells. (**C**) LA decreased the levels of ICAM-1 in BEAS-2B cells activated with TNF-α/IL-4. Three independent experiments were analyzed, and data were presented as mean ± SEM. * *P* < 0.05, ** *P* < 0.01 compared to BEAS-2B cells stimulated with TNF-α and IL-4.

BEAS-2B cells treated with licochalcone A and stimulated with 10 ng/mL TNF-α/ IL-4 had significantly reduced levels of IL-6, IL-8, CCL5, CCL11, CCL24, and MCP-1 compared to TNF-α/IL-4 activated BEAS-2B cells (Figure 9). Licochalcone A also suppressed THP-1 monocyte cell adhesion compared to TNF-α/IL-4-activated BEAS-2B cells (Figure 10A,B). In addition, licochalcone A significantly reduced ICAM-1 levels in TNF-α/IL-4-activated BEAS-2B cells (Figure 10C).

### 3.10. Effect of Licochalcone A on ROS Production

**Figure 11:**
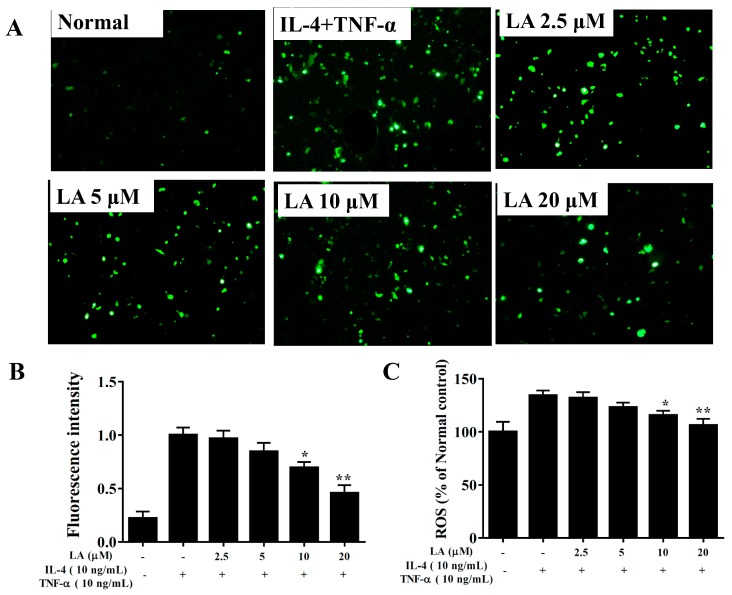
Effects of licochalcone A (LA) on ROS production in activated BEAS-2B cells. (**A**) Fluorescence microscopy images of intracellular ROS. (**B**) Fluorescence intensity of intracellular ROS. (**C**) Percentages of ROS detected in TNF-α/IL-4-activated BEAS-2B cells in the absence or presence of LA compared to untreated cells (Normal). Three independent experiments were analyzed, and data were presented as mean ± SEM. * *P* < 0.05, ** *P* < 0.01 compared to BEAS-2B cells stimulated with TNF-α and IL-4.

Fluorescence microscopy showed that licochalcone A attenuated intracellular ROS expression compared to TNF-α/IL-4-stimulated BEAS-2B cells (Figure 11A,B). In BEAS-2B cells stained with DCFH-DA, ROS production was detected using a multimode microplate reader, showing that licochalcone A decreased ROS levels in TNF-α/IL-4-stimulated BEAS-2B cells (Figure 11C).

## 4. Discussion

Licochalcone A is a natural product with multiple biological functions, including anti-inflammatory, antioxidant, antiobesity, and antitumor effects [12]. Many studies have found that licochalcone A inhibits proliferation, inducing apoptosis and autophagy effects in lung, breast, and liver cancer cells [37,38]. Licochalcone A can inhibit the inflammatory response of macrophages and reduce the secretion of inflammatory cytokines [13]. In mice with acute lung injury, licochalcone A reduces neutrophil infiltration and inflammatory cell cytokine secretion, and it promotes SOD expression. Moreover, licochalcone A is thought to have a good effect in reducing inflammation and enhancing oxidative protection [39]. Previous studies have shown that 50 mg/kg licochalcone A reduces the levels of Th2-associated cytokine in the BALF and decreases OVA-specific IgE expression in serum [18]. However, whether licochalcone A improves lung inflammation and has an antioxidation effect in asthmatic mice is not clear.

We conducted a seven-day animal toxicity test for licochalcone A, finding a 33% mortality rate for 50 mg/kg licochalcone A (data not shown). We also found that mice treated with 50 mg/kg licochalcone A via intraperitoneal injection had significantly reduced mobility and appetite compared to normal mice and mice treated with 10 mg/kg licochalcone A (data not shown). Therefore, this experiment utilized 5 or 10 mg/kg licochalcone A to explore whether the low dose of licochalcone A could improve the pathological manifestations of asthma in OVA-sensitized asthmatic mice. We also evaluated the molecular mechanism of licochalcone A in reducing inflammation of the airways and oxidative stress in asthmatic mice.

Chronic inflammation and oxidative stress are pathological manifestations characterized by the activation of persistent inflammatory cells and the release of various inflammatory mediators that cause cell and tissue destruction [40]. Therefore, sustained oxidative stress is thought to play an important role in the development of many chronic diseases, including cancer, cardiovascular disease, and chronic obstructive pulmonary disease [14,40]. An asthma attack is an acute allergic and inflammatory response, but the lung damage in patients is often caused by long-term inflammation and oxidative damage [34,36]. Previous studies pointed out that excessive oxidative stress aggravates sputum production in the airways and damages respiratory cells [32]. In asthmatic animals, ROS stimulated the activation of airway epithelial cells to release more chemokines, attracting inflammatory immune cells to infiltrate the airways and lungs [10,41]. These inflammatory immune cells would also release more inflammatory mediators, causing oxidative stress and damaging lung cells [9]. To understand whether licochalcone A improves the oxidation response of airway epithelial cells, we stimulated BEAS-2B cells to investigate ROS levels and the inflammatory response. The results showed that licochalcone A had the ability to reduce ROS levels in inflammatory BEAS-2B cells and reduce the expression of chemokines CCL11 and CCL24, reducing the activation of eosinophils in the lungs. In addition, licochalcone A inhibited MCP-1 and IL-8, reducing the migration of neutrophils and macrophages. We believe that licochalcone A has the ability to inhibit the release of more ROS from inflammatory airway epithelial cells and reduces inflammation-related chemokine secretion, reducing ROS and cell damage.

Glutathione, catalase, and superoxide dismutase are antioxidant enzymes that can reduce the damage of oxidative stress in the lungs of asthma patients [7,42], and MDA is the most abundant reactive aldehyde in lipid peroxidation, which is an important signal molecule for cell damage and can be used as an indicator of oxidative stress in cells and tissues [43]. In this study, licochalcone A had the ability to reduce MDA expression and increase GSH production, suggesting that licochalcone A regulated and improved lung peroxidation in asthmatic mice. Previous research demonstrated that licochalcone A improves acetaminophen-induced hepatotoxicity by promoting the Nrf2 antioxidant pathway [44]. Licochalcone A could increase radical-scavenging to reduce Aβ aggregation, improving Alzheimer’s disease [45]. Licochalcone A also attenuates angiotensin II-induced abdominal aortic aneurysm in mice by modulating miR-181b/SIRT1/HO-1 signaling [46]. In the current study, licochalcone A also promoted nuclear Nrf2 production, increasing HO-1 expression in the lungs of asthmatic mice. Thus, licochalcone A had protective potential against oxidation stress to maintain lung function in asthmatic mice.

ROS can also cause cell damage in the lungs. The lungs are in a high ROS environment for a long time, which causes DNA breakage in lung cells and induces cell apoptosis, reducing lung function [34]. Previous studies found that house dust mites induce inflammation, oxidative damage, DNA double-strand breaks, and cell apoptosis in the lungs of asthmatic mice [36]. In this study, we used OVA to induce asthmatic symptoms in mice and found that it could induce ROS in the lungs. However, using fluorescent γH2AX to observe DNA fragmentation, there was no γH2AX fluorescent signal in the lungs of OVA-sensitized asthmatic mice. Previous studies found that OVA-induced asthma by tracheal invasion can increase 8-isoprostane and 8-hydroxy-2’-deoxyguanosine in BALF, causing γH2AX expression in the lungs [35]. However, we induced asthma by the 2% OVA aerosol method, which seemed to only induce ROS and not DNA damage in the lungs.

To detect DNA damage in the lungs, we used CC10 as the control for the trachea, but we found that the epithelial cells of the trachea in our asthmatic model could express a great amount of CC10 compared to normal mice. Clara cells of the tracheal epithelium were stimulated by the cytokines of Th2 cells, and clara cells differentiated into goblet cells secreting a large amount of mucus and the anti-inflammatory protein CC10, which reduced airway inflammation via feedback regulation [47]. Therefore, our asthmatic mice are in the stage of continuous airway inflammation and expressed high levels of IL-6 and TNF-α in the lungs and BALF, respectively. We thought that OVA-sensitized asthmatic mice stimulated the differentiation of clara cells into goblet cells via the release of Th2 cytokines. PAS staining also revealed more goblet cell hyperplasia in the trachea, which not only released a large amount of mucus but also caused airway obstruction. Previous studies have found that IL-4 and IL-13 can stimulate goblet cell proliferation in the trachea [6]. Licochalcone A had the ability to reduce goblet cell hyperplasia, reducing excessive secretion of mucus and preventing airway obstruction and asphyxia by blocking expression of IL-4 and IL-13 in BALF and lung tissue. Interestingly, licochalcone A has the ability to reduce airway inflammation; therefore, it is not necessary for goblet cells to secrete more CC10 against inflammation in asthmatic mice.

Clinically, AHR is an important indicator for assessing lung function. Air flow and respiratory rate can be detected to evaluate the pathological characteristics and respiratory system of asthma patients [48]. Previous studies found that oxidative stress and inflammatory mediators can stimulate excessive AHR and cause deterioration of lung function [15,49]. Airway narrowing and dyspnea are the most common symptoms, and the result increases airway resistance and excessive expansion in the lungs of asthmatic patients [50]. However, the connective tissue of the airways in chronic asthma patients was elastically hardened or thickened, which decreased the elasticity of the airways and decreased alveolar surface tension [3]. Therefore, lung compliance is deteriorated in asthmatic patients. Using the intubation mode to detect AHR, licochalcone A reduced airway resistance and increased the dynamic lung compliance of asthmatic mice. Licochalcone A had a great effect on improving the physiological respiratory function of asthmatic mice. Previous studies demonstrated that high levels of IL-13 in the lungs of asthmatic patients aggravated AHR [6]. In licochalcone A-treated asthmatic mice, IL-13 expression in the lungs was significantly decreased. Therefore, our result confirms that licochalcone A reduces AHR mainly by inhibiting IL-13 expression.

There is a large amount of eosinophil infiltration in the lungs and airways of asthma patients [8]. Many studies have shown that Th2 cells in the lungs of asthma patients release excessive IL-5, causing differentiation of bone marrow cells to mature eosinophils [31]. Inflamed respiratory epithelial cells also release high levels of chemokines that attract eosinophils into the lungs [47]. Previous studies have demonstrated that activated eosinophils in the lungs release major base proteins to stimulate mast cell degranulation, causing severe allergic reactions in asthmatic patients [4]. Eosinophils also release eosinophil cationic protein and eosinophil peroxidase, causing inflammation and damage to alveolar cells [51]. Thus, excessive activated eosinophil infiltration in the lungs would affect lung function in asthmatic patients. Our experiments show that BALF and lungs of asthmatic mice have high levels of IL-5. In addition, THP-1-simulated immune cells adsorb inflamed airway epithelial cells, and licochalcone A had the ability to reduce the expression of ICAM-1 in inflamed tracheal epithelial cells, reducing THP-1 cell adhesion. Therefore, licochalcone A significantly inhibited the expression of IL-5 in the airways and reduced the inflammation of tracheal epithelial cells, inhibiting eosinophil migration and infiltration in the lung tissue and reducing the allergy and peroxidation effect.

Collagen deposition could cause lung fibrosis, which is a severe airway disease characterized by epithelial cell inflammation and injury [52]. In asthmatic patients, BALF had higher TGF-β levels, and the lung tissue significantly increased fibrosis [53]. Clinically, the forced expiratory volume in one second (FEV1) was also higher in asthma patients with lung fibrosis than patients without fibrosis [52]. We demonstrated that licochalcone A significantly inhibited collagen deposition in asthmatic mice and, therefore, would improve lung fibrosis and AHR in asthmatic mice.

IL-4 can activate B cells to secrete IgE and bind to mast cells, activating a complex of allergic reactive IgE and mast cells that release leukotrienes and histamine, causing allergic and inflammatory reactions in acute asthma patients [4]. Importantly, licochalcone A reduced IL-4 production by suppressing Th2 cell activity, improving the pathological features of asthma.

## 5. Conclusions

In conclusion, we demonstrated that licochalcone A significantly suppressed mucus secretion and eosinophil infiltration by blocking Th2 cytokine and eotaxin expression in asthmatic mice. Licochalcone A also attenuated inflammatory and oxidative responses in tracheal epithelial cells, and it ameliorated airway inflammation and oxidative stress in asthmatic mice. Our experimental results suggest that licochalcone A is a natural product and has excellent potential to ameliorate asthmatic inflammation and oxidative stress.

## Figures and Tables

**Table 1:** Primers used in real-time PCR analysis of cytokine and chemokine mRNA expression.

Gene	Forward Primer	Reverse Primer	Gene Number
*COX-2*	ACCAGCAGTTCCAGTATCAGA	CAGGAGGATGGAGTTGTTGTAG	NM_011198
*MUC5AC*	AATGCTGGTGCCTGTGTCT	CCTCCTATGCCATCTGTTGTG	NM_017511
*IL-6*	AGGACCAAGACCATCCAATTCA	GCTTAGGCATAACGCACTAGG	NM_031168
*IFN-* *γ*	CAGCAACAACATAAGCGTCATT	ACCTCAAACTTGGCAATACTCA	NM_000619.2
*IL-* *13*	GCTCCAGCATTGAAGCAGTG	CGTGGCAGACAGGAGTGTT	NM_008355.3
*IL-5*	ATCCTCCTGCCTCCTCTTCC	GGTTCCATCTCCAGCACTTCA	NM_000879
*IL-* *4*	TCCGTGCTTGAAGAAGAACTC	GTGATGTGGACTTGGACTCATT	NM_021283.2
*CCL24*	AGGCAGTGAGAACCAAGT	GCGTCAATACCTATGTCCAA	NM_019577.4
*CCL11*	GGCTTCATGTAGTTCCAGAT	CCATTGTGTTCCTCAATAATCC	NM_011330.3
*β-actin*	AAGACCTCTATGCCAACACAGT	AGCCAGAGCAGTAATCTCCTTC	NM_007393.3
